# The Role of CaMKII Overexpression and Oxidation in Atrial Fibrillation—A Simulation Study

**DOI:** 10.3389/fphys.2020.607809

**Published:** 2020-12-18

**Authors:** Wei Wang, Weijian Shen, Shanzhuo Zhang, Gongning Luo, Kuanquan Wang, Yong Xu, Henggui Zhang

**Affiliations:** ^1^Shenzhen Key Laboratory of Visual Object Detection and Recognition, Harbin Institute of Technology, Shenzhen, China; ^2^Peng Cheng Lab, Shenzhen, China; ^3^Biological Physics Group, School of Physics and Astronomy, The University of Manchester, Manchester, United Kingdom; ^4^Department of Computer Science and Technology, Harbin Institute of Technology, Harbin, China

**Keywords:** atrial fibrillation, calcium cycling, CaMKII overexpression, CaMKII oxidation, cardiac modeling

## Abstract

This simulation study aims to investigate how the Calcium/calmodulin-dependent protein kinase II (CaMKII) overexpression and oxidation would influence the cardiac electrophysiological behavior and its arrhythmogenic mechanism in atria. A new-built CaMKII oxidation module and a refitted CaMKII overexpression module were integrated into a mouse atrial cell model for analyzing cardiac electrophysiological variations in action potential (AP) characteristics and intracellular Ca^2+^ cycling under different conditions. Simulation results showed that CaMKII overexpression significantly increased the phosphorylation level of its downstream target proteins, resulting in prolonged AP and smaller calcium transient amplitude, and impaired the Ca^2+^ cycling stability. These effects were exacerbated by extra reactive oxygen species, which oxidized CaMKII and led to continuous high CaMKII activation in both systolic and diastolic phases. Intracellular Ca^2+^ depletion and sustained delayed afterdepolarizations (DADs) were observed under co-existing CaMKII overexpression and oxidation, which could be effectively reversed by clamping the phosphorylation level of ryanodine receptor (RyR). We also found that the stability of RyR release highly depended on a delicate balance between the level of RyR phosphorylation and sarcoplasmic reticulum Ca^2+^ concentration, which was closely related to the genesis of DADs. We concluded that the CaMKII overexpression and oxidation have a synergistic role in increasing the activity of CaMKII, and the unstable RyR may be the key downstream target in the CaMKII arrhythmogenic mechanism. Our simulation provides detailed mechanistic insights into the arrhythmogenic effect of CaMKII overexpression and oxidation, which suggests CaMKII as a promising target in the therapy of atrial fibrillation.

## Introduction

Atrial fibrillation (AF) is the most common persistent arrhythmia, affecting ~33 million of the world's population (Chugh et al., 2014), while the treatment of AF is difficult due to its self-reinforcing and structural remodeling properties. Therefore, the mechanistic understanding of AF becomes necessary. Recent studies suggest that Ca^2+^-deregulation plays an important role in atrial fibrillation, which may be linked by the Calcium/calmodulin (Ca^2+^/CaM)-dependent protein kinase II (CaMKII) (Heijman et al., 2014). CaMKII is a multifunctional protein kinase widely expressed in the heart, which can phosphorylate and regulate the functions of many substrate proteins in myocytes. The CaMKII dependent phosphorylation of L-type Ca^2+^ channel (LTCC) can promote the opening of LTCC and slower the channel inactivation process (Xiao et al., 1994), leading to larger L-type Ca^2+^ current (I_CaL_) density. The phosphorylation of ryanodine receptor (RyR) by CaMKII can increase the sensitivity of RyR to Ca^2+^, thereby RyR would have a closer connection with the intracellular concentration of Ca^2+^ ([Ca^2+^]_i_) (Wehrens et al., 2004). The CaMKII dependent phosphorylation of phospholamban (PLB) increases the affinity of SR Ca^2+^-ATPase (SERCA) for Ca^2+^, leading to an enhancement of its Ca^2+^ transportation rate (Odermatt et al., 1996). Particularly in atria, sarcolipin can undergo CaMKII dependent phosphorylation, which also influences SERCA and results in an increased SR uptake (Heijman et al., 2014). Besides these targets directly related to calcium cycling, CaMKII also regulates other membrane currents, including I_Na_, I_NaL_, I_Kur_, I_to_, I_K1_, I_NCX_, etc. (Tessier et al., 1999; Maltsev et al., 2008; Wagner et al., 2009). The above-mentioned CaMKII regulation of downstream target proteins enables cardiomyocytes to adaptively enhance the speed of intracellular Ca^2+^ circulation when the heart rate increases, thereby continuously and effectively contracting at a higher heart rate.

However, increased CaMKII activity and AF have been found to have a mutual promoting effect. On the one hand, CaMKII expression and activity increases were observed in various species with atrial tachycardia and fibrillation, such as human with chronic AF (Neef et al., 2010; Voigt et al., 2012), goat with long-standing AF (Greiser et al., 2009) and canine with pacing-induced atrial tachycardia remodeling (Wakili et al., 2010), showing the role of AF in promoting CaMKII overexpression and hyperactivities. On the other hand, CaMKII abnormities were considered to promote ectopic activities such as early afterdepolarizations (EAD) (Qi et al., 2009) and delayed afterdepolarizations (DAD) (Dobrev et al., 2011), and to improve reentry generations by increasing the repolarization dispersion (Yue et al., 2011) or slowing the conduction velocity of the electrical wave (Wagner et al., 2006; Wang et al., 2018), all of which were the key mechanisms related to AF. In addition, AF was found to relate to a higher level of oxidative stress and CaMKII oxidation also increased in AF patients. Purohit et al. (2013) have shown a direct link between oxidative CaMKII activation and AF by using MMVV mice, which presented knock in mice without oxidation sites in CaMKII were failed to induce AF induction with Angiotensin II infusion. Increased excessive reactive oxygen species (ROS) in cardiomyocytes will oxidize and activate more CaMKII (Münzel et al., 2015), which may aggravate existing heart diseases.

Above evidence showed that the overexpression and oxidation of CaMKII may play an important role in AF, whereas the detailed mechanism remained insufficient elucidation. With the advancement of cardiac modeling, an appropriate cardiac model can interpret more experimental observations and help explore the underlying mechanisms and their interrelationships (Clayton et al., 2011; Wang et al., 2019; Ye et al., 2019; Luo et al., 2020). In this study, we investigated the arrhythmogenic mechanism underlying CaMKII overexpression and oxidation by using a mathematical mouse atrial model incorporated with the CaMKII overexpression and oxidation module. We demonstrated that how the CaMKII influenced downstream targets under different abnormal conditions, and how these changes further induced calcium transient (CaT) instabilities or DADs, revealing the mechanism behind CaMKII overexpression and oxidation to AF and cardiac arrhythmias.

## Method

In this study, the mouse atrial model constructed by Zhang et al. (2020) was incorporated with the CaMKII activation module developed by Morotti et al. (2014) and used as the baseline model. We further integrated a new built CaMKII oxidation module and a refitted CaMKII overexpression module into the baseline model for investigating the role of CaMKII oxidation and overexpression in AF. The details of each model are described in the following part. The source code of the model is available under the request to the authors (wangwei2019@hit.edu.cn).

### CaMKII Activation Model

**Figure 1:**
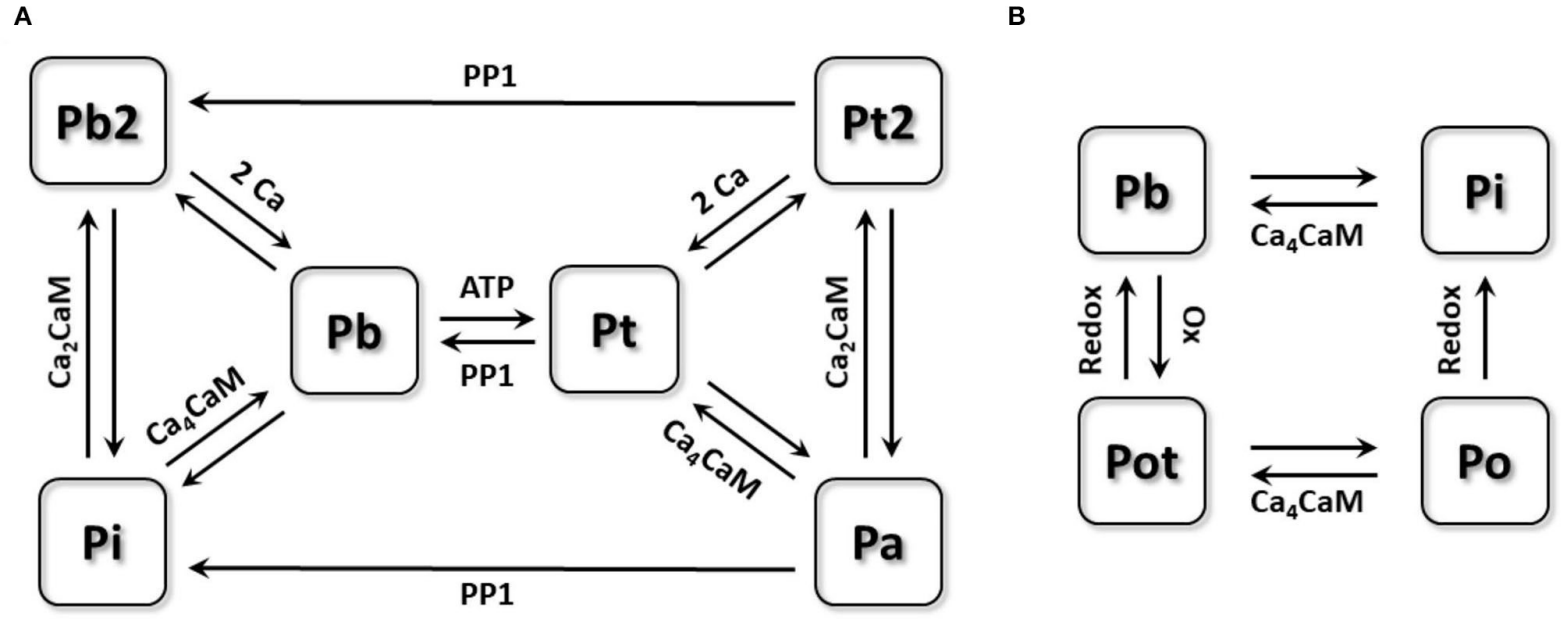
The schematic diagram of the Markov chain model of CaMKII activation model and oxidation model. **(A)** 6-state Markov chain model of CaMKII activation model. **(B)** 4-state Markov chain model of CaMKII oxidation model.

CaMKII is a multimeric enzyme assembled by 10 to 12 subunits, each of which can be activated by binding calmodulin (CaM) and further autophosphorylate adjacent subunits to maintain the activation (Saucerman and Bers, 2008). For modeling this activation process of CaMKII, the 6-state Markov chain model proposed by Saucerman and Bers (2008) as shown in Figure 1A was used. In this model, the inactivated state Pi can bind with the Ca_4_CaM (a CaM binding with 4 Ca^2+^) and transform to the active state Pb. CaMKII in this state Pb can phosphorylate neighboring subunits in the presence of ATP, transforming them to the state Pt, which process is called the autophosphorylation of CaMKII. Autophosphorylated subunits have long-lasting activity, even if they dissociate with CaMs and further transform to the state Pa. In addition, CaMKII may also bind with Ca_2_CaM in an environment of more unsaturated CaM (shown as the state Pi to Pb2 and the state Pa to Pt2), waiting for another two Ca^2+^ for activation. Finally, CaMKII under the state (Pa, Pt2) can be dephosphorylated by protein phosphatase 1 (PP1) to the state (Pi, Pb2), respectively. For the whole model, there are two inactive states (Pi, Pb2) and four active states (Pb, Pt, Pt2, Pa), and all active states are assumed to have 100% activity. Morotti et al. (2014) incorporated this CaMKII activation module into a mouse ventricular model and reimplemented a CaMKII dependent RyR phosphorylation part, which was also inherited by this study as we assume the CaMKII activation process is the same in mouse atria and ventricles.

In this study, the cell model incorporated only this CaMKII activation module was defined as the wild type (WT) model.

### CaMKII Oxidation Model

For modeling CaMKII behaviors under oxidative stress, we built a new four-state Markov chain model (Figure 1B). CaMKII under the state Pb was reported being able to be oxidized by ROS, after which CaMKII can have long-lasting activity (Erickson et al., 2008). This process is called CaMKII oxidation, which is quite similar to its autophosphorylation process. Due to the unraveled interaction between the CaMKII oxidation and auto-phosphorylation, we assumed that these two processes were conflicting with each other, which means CaMKII being autophosphorylated cannot be further oxidized. According to this assumption, only CaMKII under the state Pb can be oxidized by ROS and then transfers to the active state Pot, which can dissociate with Ca_4_CaM but still remain active (state Po). Oxidized CaMKII (state Pot and Po) can be deoxidized by methionine sulfoxide reductases (MsrA) to the inactive state (Pi and Pb), respectively. Each state transition equation can be written as:

$$\begin{array}{l} \overset{∙}{\text{Pb}}=-k_{ox}\times \frac{\left[\text{ROS}\right]}{\left[\text{ROS}\right]+K_{m_{\_}ROS}}\times \text{Pb}+{\text{k}}_{\text{redox}}\times \frac{\left[\text{MsrA}\right]}{\left[\text{MsrA}\right]+{\text{K}}_{{\text{m}}_{\_}\text{MsrA}}}\\ \;\times \text{Pot}+{\text{k}}_{\text{ib}}\times \left[{\text{Ca}}_{4}\text{CaM}\right]\times \text{Pi}-{\text{k}}_{\text{ib}}\times \text{Pb}+{\text{T}}_{\text{other}}\\ \overset{∙}{\text{Pot}}={+k}_{ox}\times \frac{\left[\text{ROS}\right]}{\left[\text{ROS}\right]+K_{m_{\_}ROS}}\times \text{Pb}-k_{redox}\times \frac{\left[\text{MsrA}\right]}{\left[\text{MsrA}\right]+K_{m_{\_}MsrA}}\\ \;\times \text{Pot}+k_{ib}\times \left[{\text{Ca}}_{4}\text{CaM}\right]\times \text{Po}-k_{bi}\times \text{Pot}\\ \overset{∙}{\text{Pi}}={+k}_{redox}\times \frac{\left[\text{MsrA}\right]}{\left[\text{MsrA}\right]+{\text{K}}_{{\text{m}}_{\_}\text{MsrA}}}\times \text{Po}\\ \;-k_{ib}\times \left[{\text{Ca}}_{4}\text{CaM}\right]\times \text{Pi}+k_{bi}\times \text{Pb}+{\text{T}}_{\text{other}}\\ \overset{∙}{\text{Po}}=-k_{redox}\times \frac{\left[\text{MsrA}\right]}{\left[\text{MsrA}\right]+K_{m_{\_}\text{MsrA}}}\times \text{Po}\\ \;-k_{ib}\times \left[{\text{Ca}}_{4}\text{CaM}\right]\times \text{Po}+k_{bi}\times \text{Pot} \end{array}$$

where T_other_ denotes all other transitions with the original model, [ROS], [MsrA], and [Ca_4_CaM] represent the concentration of ROS, MsrA and Ca_4_CaM. Details of all parameters in the equation are listed in Table 1.

**Table 1:** Parameters in the CaMKII oxidation model.

**Parameter**	**Value**	**Unit**
k_bi_	2.2	s^−1^
k_ib_	2.2/(33.5 × 10^−3^)	μM^−1^s^−1^
k_ox_	6.48 × 10^−6^	s^−1^
K_m_ROS_	60	μM
K_m_MsrA_	0.34	mM
k_redox_	0.28	s^−1^

**Figure 2:**
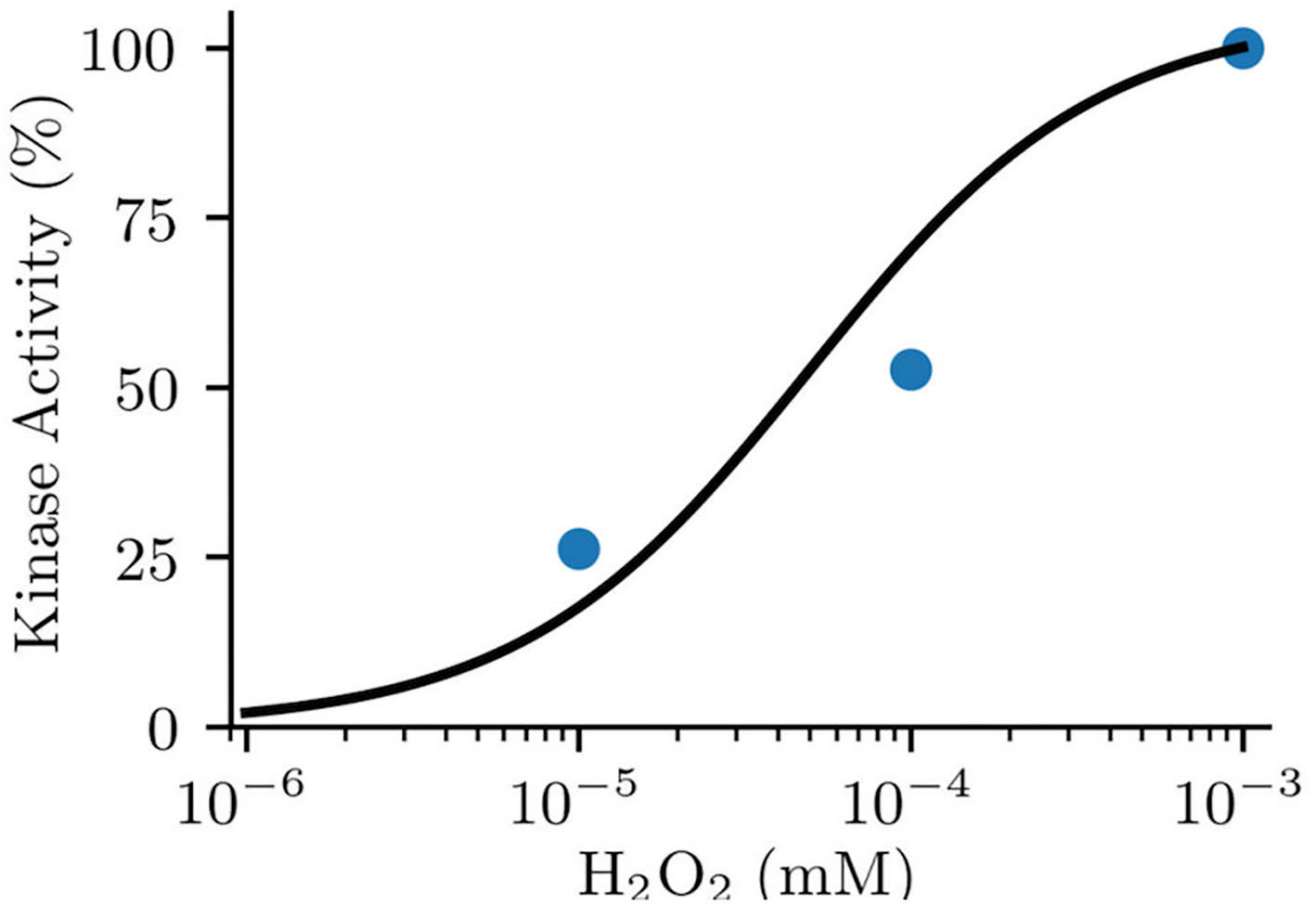
Fitted model data (line) to experimental data (dot) of normalized kinase activity of CaMKII by oxidative activation.

Parameters including k_bi_ and k_ib_ were inherited from the original model (Saucerman and Bers, 2008). The enzyme activity of MsrA including the reduction rate (k_redox_) and the Michaelis-Menten constant of MsrA (K_m_MsrA_) were used as those measured in experiments (Kim and Gladyshev, 2005). For deciding the oxidation rate (k_ox_) and the Michaelis-Menten constant of ROS (K_m_ROS_), we replicated the Erickson et al. (2008)'s experiment, which presented the relationship between the kinase activity of CaMKII and various concentration of ROS (shown in Figure 2). Considering the similar electrophysiology of the mouse atrium and ventricle, we also incorporated the oxidation module into the mouse ventricular model (Morotti et al., 2014) for investigating the chamber-specificity of oxidative CaMKII arrhythmogenicity in mouse. More details about this can be found in Supplementary Document 1.

In this study, the CaMKII oxidation model was activated by adding ROS. As normal ROS level can reach about 35 μM and increase as much as 100 times under oxidative stress (Foteinou et al., 2015), we applied 200 μM ROS to the model for mimicking a pathophysiological condition.

### CaMKII Overexpression Model

In this study, we increased the total concentration of intracellular CaMKII to 6-fold of the WT model for modeling its overexpression as suggested by the previous study (Zhang et al., 2003; Morotti et al., 2014). We named this model the CaMKII-OE model. Since the main downstream proteins influenced by CaMKII including LTCC, RyR, and PLB had been dynamically linked to the total amount of activated CaMKII, i.e., the phosphorylation levels of the above three proteins will change with the increase of CaMKII concentration, they were not modified independently again. Besides, other membrane currents, which were reported influenced by CaMKII overexpression, but did not directly connect with the concentration of CaMKII in the model, were modified separately by refitting the experimental data (Maier et al., 2003; Wagner et al., 2006, 2009, 2011; Maltsev et al., 2008) to reproduce the CaMKII overexpression effect. In total, these currents include late-sodium current (I_NaL_), transient outward potassium current (I_to_), inward rectifier potassium current (I_K1_) and Na^+^-Ca^2+^ exchanger current (I_NCX_). Details of modifications on these membrane currents were summarized in Table 2.

**Table 2:** Changes of in the CaMKII-OE model based on experimental data.

**Target current**	**Variations in the CaMKII-OE model**
I_NaL_	Shifting its inactivation curve toward the positive potential by 6.8 mV
I_to_	Multiplying the transition rate of K_f_to_ by 5-fold
I_K1_	Reducing the maximum conductance by 40%
I_NCX_	Increasing the maximum conductance by 30%

**Figure 3:**
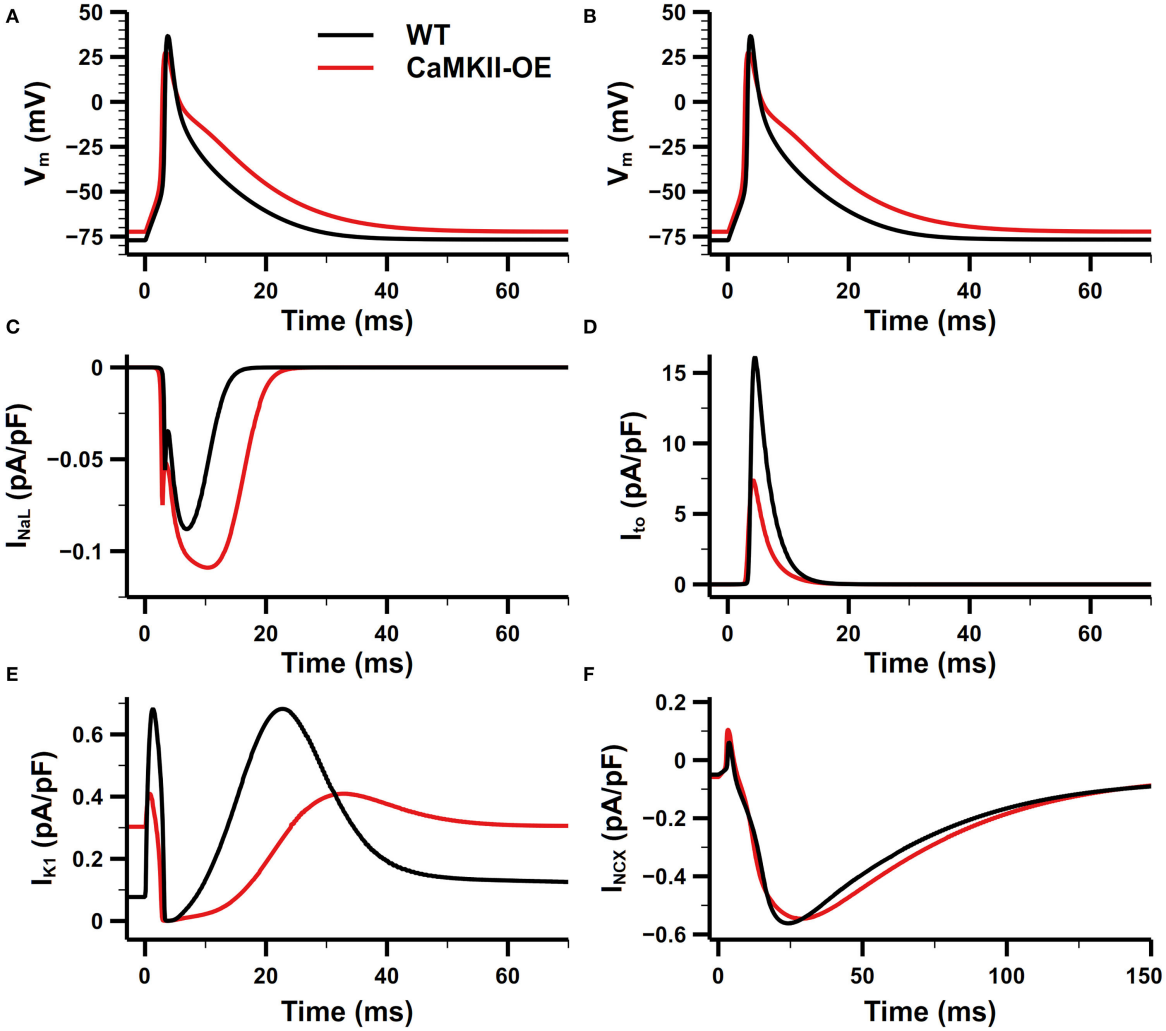
Comparison of the action potential (AP) and main membrane currents between CaMKII-OE and WT model. **(A,B)** Action potential, **(C)** late-sodium current (INaL), **(D)** transient outward potassium current (Ito), **(E)** inward rectifier potassium current (IK1), **(F)** Na+-Ca^2+^ exchanger current (INCX).

The comparison of the action potential (AP) and main membrane currents between the CaMKII-OE and WT model is shown in Figure 3. Experimental data have shown that the CaMKII overexpression can lead to increased current density of I_NaL_ (Wagner et al., 2011) and shift the steady-state inactivation of the channel to more positive voltage (Maltsev et al., 2008). In this model, we achieved this variation by shifting the inactivation curve of I_NaL_ 6.8 mV toward the positive potential. Compared with the WT model, the peak current density of I_NaL_ during the action potential has increased by ~50% (Figure 3C), consistent with experimental data (Wagner et al., 2011). Physiological experiments also found that I_to_ showed accelerated recovery from inactivation, a more negative steady-state inactivation curve, and smaller current density under the CaMKII-OE condition (Wagner et al., 2009). We increased the rate constant that control the transition of I_to_ from the closed and open states to their corresponding inactivated states to its 5 times to reproduce this observation. As shown in Figure 3D, I_to_ in the CaMKII-OE model reached an ~60% decrease in current amplitude measured in experiments (Wagner et al., 2009). The maximum conductance of I_K1_ was decreased by 40% to fit the experimental data (Wagner et al., 2009). Simulation results of the model showed (Figure 3E) that the peak current amplitude of I_K1_ in the CaMKII-OE model decreased significantly, whereas I_K1_ increased during the resting period due to an increased resting potential of the model. Meanwhile, it is worth noting that although the maximum conductance of I_NCX_ was increased by 30% in the CaMKII-OE model to replicate the influence of CaMKII (Maier et al., 2003), I_NCX_ itself did not change much at the steady state (Figure 3F), which was mainly due to the effect of CaMKII overexpression on intracellular ion concentrations.

### Simulation Protocols

In this study, the steady-state protocol was used to investigate the influence of CaMKII overexpression and oxidation on the action potential and intracellular calcium cycling. The cell model was paced for around 5 min (cell time) at 1 Hz until the steady state was reached. The steady state was defined as the situation when differences in ion concentrations between two consecutive beats were lower than one hundred thousandths of the basal level.

To further study the effect of abnormal CaMKII at higher heart rates, the widely used burst pacing protocol was applied to induce abnormal cell behaviors. The cell model was first initialized with 1-Hz pacing, then stimulated at 10 Hz for 12 s (burst pacing period). Finally, we resumed the stimulation frequency to 1 Hz to see how myocytes react.

We also carried out a series of clamping simulations to investigate the RyR property, in which we recorded its release amplitude and activation threshold at different sarcoplasmic reticulum (SR) calcium concentration ([Ca^2+^]_SR_) and phosphorylation levels. The cell model was first initialized with 1-Hz pacing, then the stimulus ceased and the [Ca^2+^]_SR_ and CaMKII dependent RyR phosphorylation level were clamped at different levels [ranged from 200 to 800 μM for [Ca^2+^]_SR_, and from 10% to 80% for RyR phosphorylation] for 10 s. After that, the clamp on [Ca^2+^]_SR_ was released and 200 stimuli at 1-Hz were applied to the model, the amplitude of RyR release of the first beat was recorded for corresponding [Ca^2+^]_SR_ and RyR phosphorylation levels. A similar clamp protocol was also used for obtaining the RyR activation threshold, whereas the differences are: (1) we substituted the I_CaL_ with a manually constructed calcium current (named I_Ca_) in order to control the transmembrane calcium influx; (2) no stimulus current was applied since the RyR could be activated purely by Ca^2+^ influx. The I_Ca_ had a square waveform with a 5-ms duration. The RyR was tested by increasing the amplitude of I_Ca_, until at least 1/4 Ca^2+^ in the SR was released, where this amplitude of I_Ca_ was defined as the RyR activation threshold. The RyR activation threshold was recorded under various levels of [Ca^2+^]_SR_ (from 200 to 800 μM) and RyR phosphorylation (from 10 to 80%) to produce a RyR activation threshold map.

## Results

### Role of CaMKII Overexpression and Oxidation in Action Potential and Calcium Cycling

**Figure 4:**
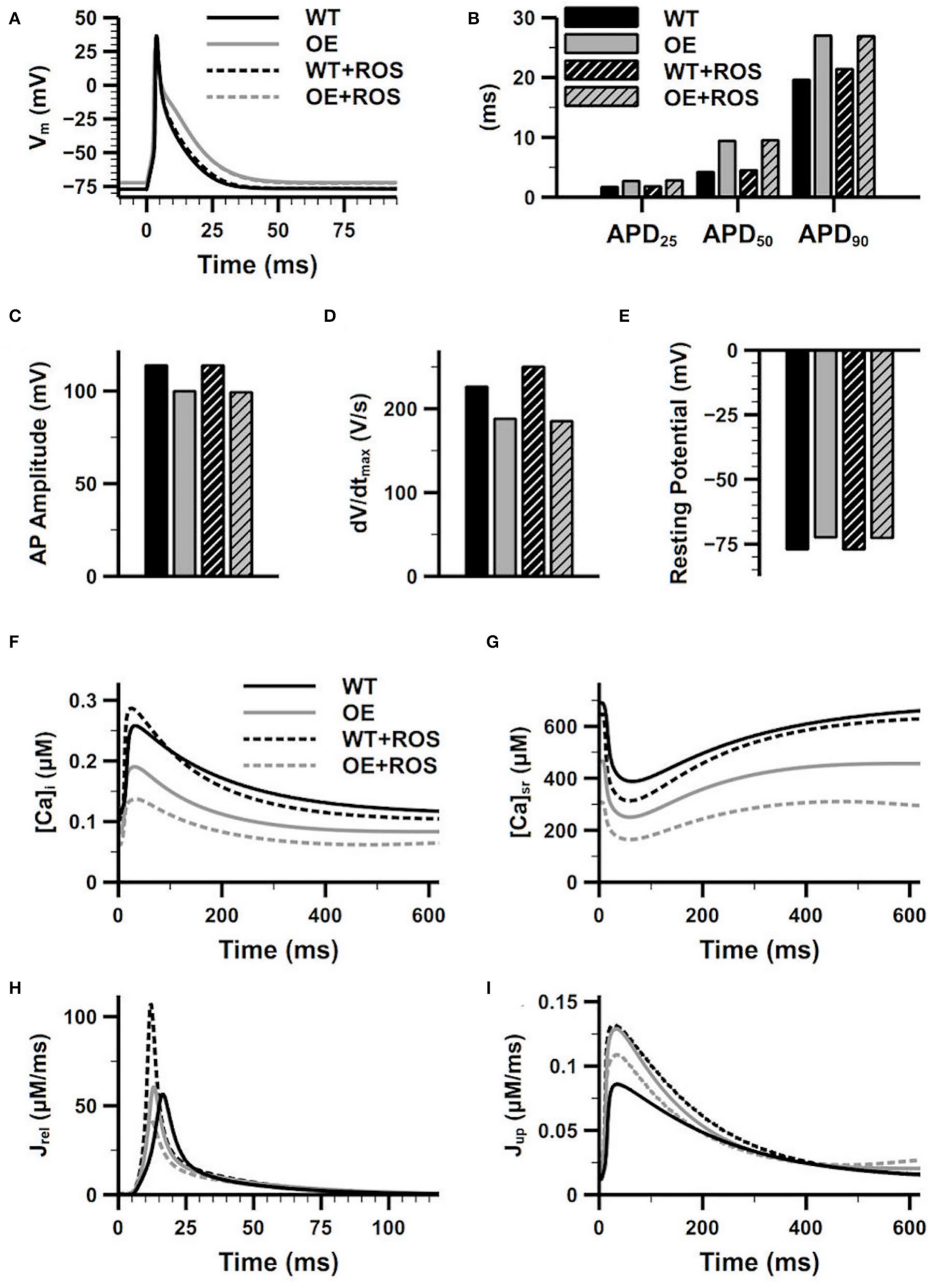
Effects of overexpression and oxidation of CaMKII on the characters of action potential (AP) and the calcium cycling process. Figure shows the superimposed AP **(A)**, recorded APD_25_, APD_50_ and APD_90_ **(B)**, AP amplitude **(C)**, dV/dt_max_ **(D)** and resting potential **(E)**, intracellular Ca^2+^ concentration **(F)**, sarcoplasmic reticulum (SR) Ca^2+^ concentration **(G)**, the SR Ca^2+^ release current **(H)**, the SR Ca^2+^ reuptake current **(I)** of the WT model and the CaMKII-OE model with and without additional ROS.

We have recorded the steady-state APs for the WT and CaMKII-OE model with and without ROS addition in Figures 4A–E. We can see significant changes on the AP morphology under the CaMKII overexpression condition. The APD is prolonged in the CaMKII-OE model, not only in APD_90_ but also APD_50_ and APD_25_ (Figure 4B). Meanwhile, a decreased AP amplitude (Figure 4C) and dV/dt_max_ (Figure 4D), and a slightly increased resting potential (Figure 4E) can be observed. The prolongation in APD_25_ and APD_50_ is not surprising since the I_to_, which contributes in the early repolarization period, substantially decreased in the CaMKII-OE model. And due to the decrease of I_K1_ and increase of I_NaL_, the increase in APD_90_ is also obvious. These changed AP characteristics manifest that CaMKII overexpression can influence the whole cardiac cycle of the AP. However, no significant impact of ROS can be observed on the AP in neither the WT nor the CaMKII-OE model, which suggests that CaMKII oxidation has limited influence on membrane currents.

On the other hand, CaMKII abnormality had a more apparent impact on calcium cycling, whose related proteins including LTCC, SERCA and RyR are the main downstream targets of CaMKII. As shown in Figures 4F,G, both the [Ca^2+^]_i_ and [Ca^2+^]_SR_ are lower in the CaMKII-OE model compared with the WT model during the whole cardiac cycle, manifesting there was intracellular Ca^2+^ depletion under CaMKII overexpression. Interestingly, the SR Ca^2+^ uptake current increased with CaMKII overexpression (J_up_, Figure 4I), which should be smaller with decreased [Ca^2+^]_i_. This was because the CaMKII dependent phosphorylation of PLB led to a quicker Ca^2+^ transportation rate through SERCA, and this influence was larger than that caused by decreased [Ca^2+^]_i_ itself. On the other hand, no significant variation of CaT amplitude was found (Figure 4F), and the increase of the SR Ca^2+^ release current (J_rel_) was also ignorable (Figure 4H). After the ROS application, oxidized CaMKII augmented the CaT compared with the WT model, manifesting as a larger CaT amplitude and a faster [Ca^2+^]_i_ increasing and decreasing rate (Figure 4F), which was mainly due to the increase of J_rel_ and J_up_ (Figures 4H,I). However, the opposite situation was found in the CaMKII-OE model. CaMKII oxidation decreased J_rel_ and J_up_, which may be attributed to the Ca^2+^ depletion caused by CaMKII overexpression. As a result, [Ca^2+^]_i_ and [Ca^2+^]_SR_ further decreased, and the CaT amplitude also declined (Figures 4F,G).

### Role of CaMKII Overexpression and Oxidation in DAD and AF

**Figure 5:**
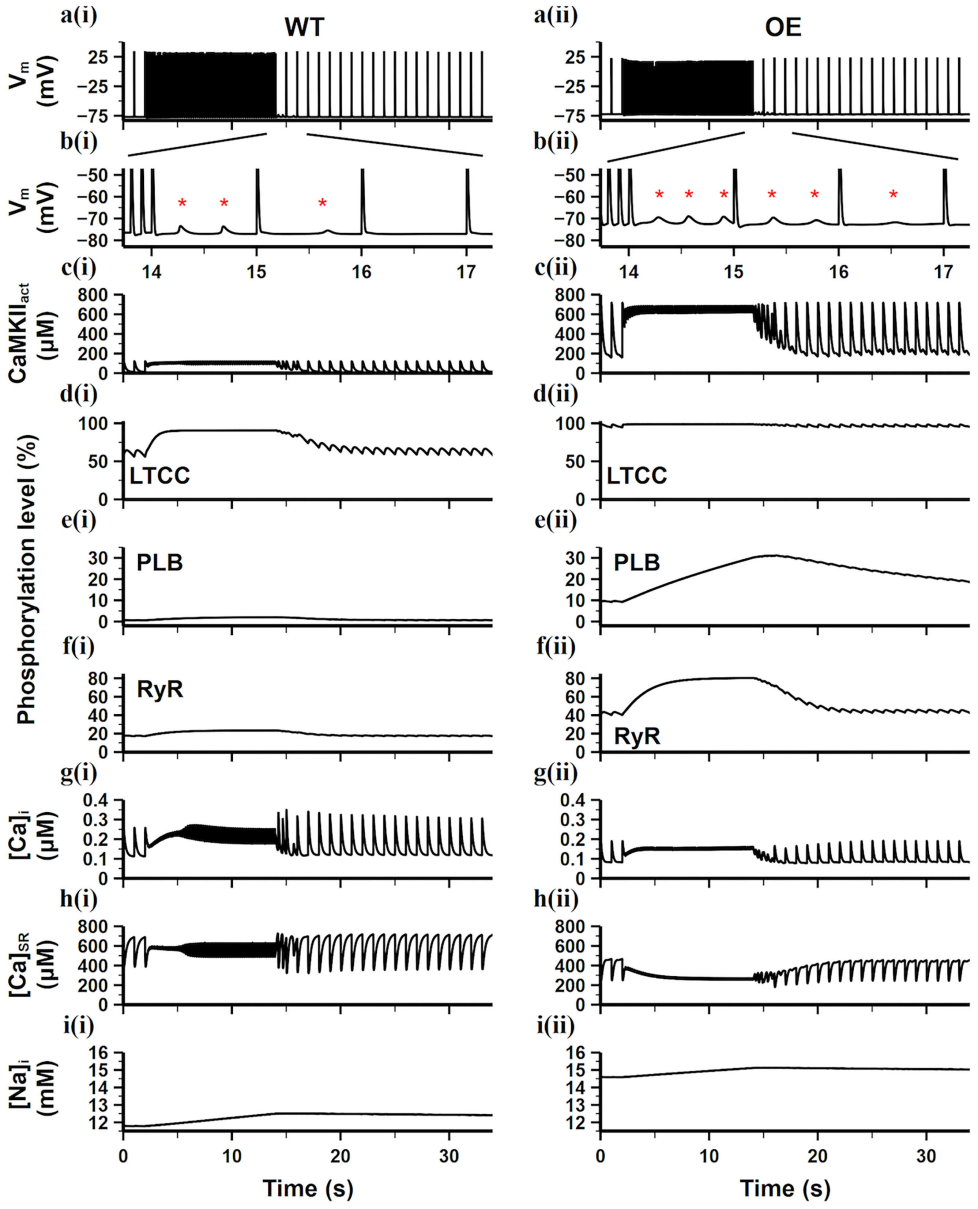
Simulation results of the WT model [**a(i)**–**i(i)**] and the CaMKII-OE model [**a(ii)**–**i(ii)**] under the burst pacing protocol. **(a)** recorded AP during the whole simulation time; **(b)** enlarged AP record after burst pacing (from time point 13.5–17.5 s); **(c)** the concentration of activated CaMKII in cell junctional area; **(d–f)** the phosphorylation level of LTCC **(d)**, PLB **(e)** and RyR **(f)**; **(g)** intracellular Ca^2+^ concentration; **(h)** sarcoplasmic reticulum (SR) Ca^2+^ concentration; **(i)** intracellular Na^+^ concentration. Generated DADs are marked by “*”.

To further study the effects of CaMKII overexpression and oxidation on DAD generation and AF, simulations in this section used the burst pacing protocol described in section Simulation Protocols. The simulation results of the WT and CaMKII-OE model were compared in Figure 5. We can see before the fast pacing (from time point 0 to 2 s), the activity level of CaMKII was significantly higher in the CaMKII-OE model, which presented around 160 μM activated CaMKII (CaMKII_act_) during the diastolic phase and about 700 μM during the systolic phase (Figures 5ci,ii). Accordingly, the downstream targets of CaMKII including LTCC, PLB and RyR were phosphorylated to higher levels. Specifically, CaMKII overexpression led to nearly 100% phosphorylation of LTCC, 10 times phosphorylation of PLB and doubled phosphorylation of RyR compared with the WT model, respectively (Figures 5ei,ii–gi,ii). Both [Ca^2+^]_i_ and [Ca^2+^]_SR_ were lower in the CaMKII-OE model (Figures 5gi,ii,hi,ii). Meanwhile, the steady-state intracellular Na^+^ ([Na^+^]_i_) was 3 mM higher in the CaMKII-OE model than that in the WT model (Figures 5Ii,ii).

During the fast pacing period (from 2 to 14 s), diastolic CaMKII activity was significantly increased compared with the slow pacing rate while systolic CaMKII activity almost remained the same (Figures 5ci,ii). In the WT model, since the total amount of CaMKII_act_ was at a relatively low level, only the LTCC phosphorylation level apparently increased, whereas PLB and RyR phosphorylation level grew slightly (Figures 5di–fi). On the contrary, LTCC phosphorylation in the CaMKII-OE model was already nearly 100% before the fasting pacing (Figure 5dii), therefore it cannot further increase with higher CaMKII_act_, leading to negligible changes on [Ca^2+^]_i_ influx. Meanwhile, PLB and RyR phosphorylation level increased considerably (Figures 5eii,fii), resulting in Ca^2+^ depletion in the SR content [see the [Ca^2+^]_SR_ decrease as shown in Figure 5hii].

When the pacing rate returned to 1 Hz (from 14 to 40 s), DADs appeared in the next several cardiac cycles in both models (Figures 5bi,ii). This is mainly because fast pacing stimuli led to instability of RyR and extra SR Ca^2+^ release during the diastolic period. This suddenly increased [Ca^2+^]_i_ gave rise to an abruptly enhanced forward mode of I_NCX_ (Ca^2+^ extrusion mode), therefore a depolarizing current was formed. However, this instability cannot last since the cell is adaptive to the variation in the pacing rate. In the WT model, only three DADs were seen in the next two cardiac cycles, whereas six DADs were found in the next three cardiac cycles in the CaMKII-OE model, showing the disturbing role of CaMKII overexpression in cell's adaptive ability.

**Figure 6:**
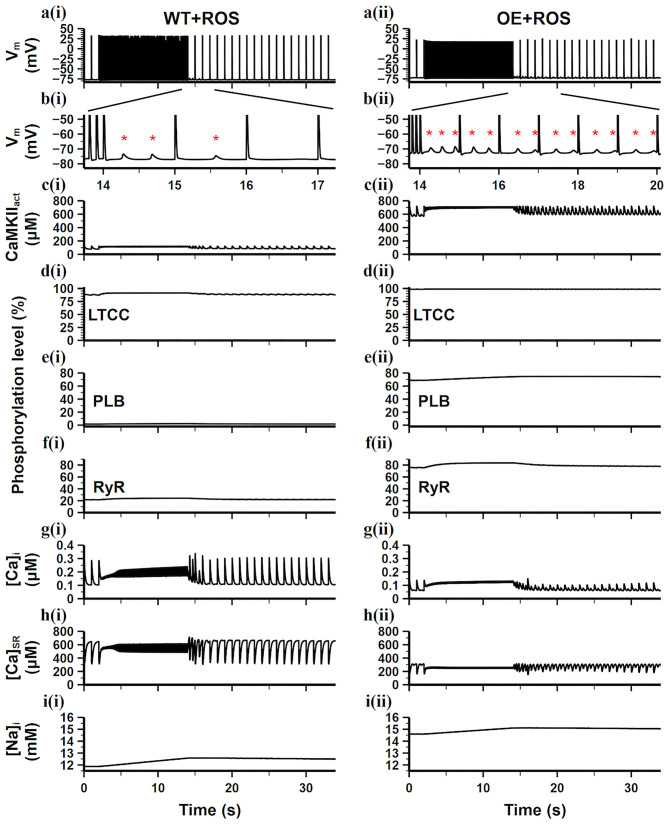
Simulation results of the WT model [**a(i)**–**i(i)**] and the CaMKII-OE model [**a(ii)**–**i(ii)**] with the application of ROS under the burst pacing protocol. **(a)** Recorded AP during the whole simulation time; **(b)** enlarged AP record after burst pacing (from time point 13.5–17.5 s); **(c)** the concentration of activated CaMKII in cell junctional area; **(d–f)** the phosphorylation level of LTCC **(d)**, PLB **(e)** and RyR **(f)**; **(g)** intracellular Ca^2+^ concentration; **(h)** sarcoplasmic reticulum (SR) Ca^2+^ concentration; **(i)** intracellular Na^+^ concentration. Generated DADs are marked by “*”.

We further added ROS into the WT and CaMKII-OE model, respectively (results are shown in Figure 6), for investigating the reaction of cardiomyocytes under oxidative stress. We found that ROS had a limited impact on the WT model. Adding ROS in the WT model only noticeably increased LTCC phosphorylation level (From 60% in Figure 5di to 87% in Figure 6di), while other targets phosphorylation varied negligibly. Consequently, ion homeostasis of the WT model retained under oxidative stress, no significant variation of [Ca^2+^]_i_, [Ca^2+^]_SR_, [Na^+^]_i_ was observed (Figures 6gi,hi). The main reason for this is that the basal amount of CaMKII in the WT model was relatively low, therefore the influence of ROS was limited since ROS mainly increased the diastolic activity of CaMKII. As a result, After the burst pacing, only three DADs presented (Figure 6bi), which was the same as the WT model, showing the adaptive ability of myocyte to ROS under the normal condition.

On the contrary, additional ROS hugely influenced the CaMKII-OE model. Due to the overexpression of CaMKII, ROS led to an increase in diastolic CaMKII_act_ to 600 μM (Figure 6cii), which was about 4.5 times of the total CaMKII in the WT model. Therefore, the phosphorylation levels of all targets including LTCC, PLB and RyR were significantly increased (all over 65% shown Figures 6dii–fii). Substantial low [Ca^2+^]_i_, [Ca^2+^]_SR_, and high [Na^+^]_i_ was observed (Figures 6gii,hii), manifesting severer Ca^2+^ depletion. As a result, more DADs presented (Figure 6bii) after the burst pacing, and lasted till the end of the simulation. Therefore, we conclude that oxidative stress could significantly disrupt the ion hemostasis under the CaMKII overexpression scenario, during which the stability of AP and CaT would decrease, which might become the origin of AF and cardiac arrhythmias.

### Influential Factors of the CaT Stability

**Figure 7:**
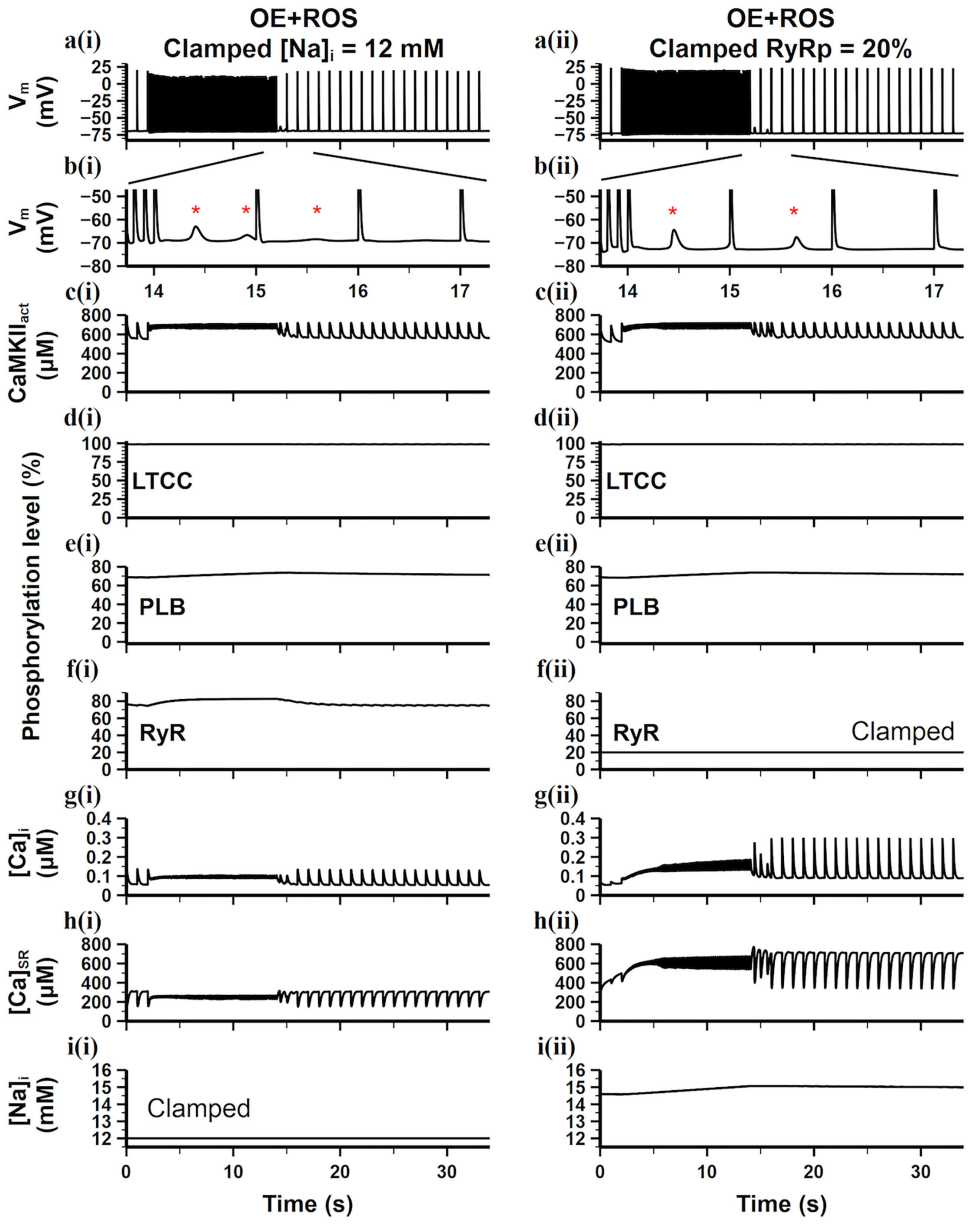
Simulation results of the CaMKII-OE model with the application of ROS under the burst pacing protocol, the [Na^+^]_i_ [**a(i)**–**i(i)**] or the level of RyR phosphorylation [**a(ii)**–**i(ii)**] in the model was clamped. **(a)** Recorded AP during the whole simulation time; **(b)** enlarged AP record after burst pacing (from time point 13.5–17.5 s); **(c)** the concentration of activated CaMKII in cell junctional area; **(d–f)** the phosphorylation level of LTCC **(d)**, PLB **(e)** and RyR **(f)**; **(g)** intracellular Ca^2+^ concentration; **(h)** sarcoplasmic reticulum (SR) Ca^2+^ concentration; **(i)** intracellular Na^+^ concentration. Generated DADs are marked by “*”.

CaT stability is determined by many factors, especially ion concentration hemostasis and Ca^2+^ handling related channel properties. Morotti et al. (2014) has shown that the CaT stability disturbed by CaMKII overexpression in mouse ventricles was closely related to the increasing [Na^+^]_i_. For further verifying if high [Na^+^]_i_ is the leading arrhythmogenic mechanism of CaMKII overexpression under oxidative stress in mouse atria, we clamped the [Na^+^]_i_ to find out its role in the CaT stability and DAD generation. We first clamped [Na^+^]_i_ at a high level [14.6 mM, the [Na^+^]_i_ level in the CaMKII-OE+ROS model, results not shown], and found that the number of generated DADs slightly decreased but still maintained, indicating that a small increase in [Na^+^]_i_ during rapid stimulation had limited effect on DAD generation. However, when we clamped the [Na^+^]_i_ at a low level of 12 mM [the [Na^+^]_i_ level in the WT model], the presence of DADs significantly decreased (Figures 7ai–ii), reflecting restored cell stability with a lower level of [Na^+^]_i_.

Moreover, CaMKII hyperactivity resulting from overexpression and oxidation increased the phosphorylation levels of downstream targets, which also has a great impact on CaT instability. For investigating the leading phosphorylation target which connects to the DAD generation, we clamped the CaMKII dependent phosphorylation levels of LTCC, PLB, and RyR, respectively, to the WT level. Results showed that only the clamp on RyR phosphorylation significantly inhibited the DAD generation (Figures 7aii–iii). We found [Ca^2+^]_i_ instability maintained in just two cardiac cycles after fast pacing and became stable quickly. Interestingly, both of the [Ca^2+^]_i_ and [Ca^2+^]_SR_ increased during the fast pacing period (Figures 7gii,hii), manifesting an increased net Ca^2+^ transmembrane influx. Therefore, the CaT amplitude also returned to the WT level quickly (Figure 7gii), presenting the high effectiveness of RyR phosphorylation inhibition. It is worth noting that the [Na^+^]_i_ in this case was still high (Figure 7iii), but it did not impair the cell stability, indicating the high [Na^+^]_i_ may not be the leading arrhythmogenic mechanism of CaMKII overexpression and oxidation in mouse atria. Instead, the diastolic SR Ca^2+^ release originated from unstable RyRs may be the direct cause of DADs.

**Figure 8:**
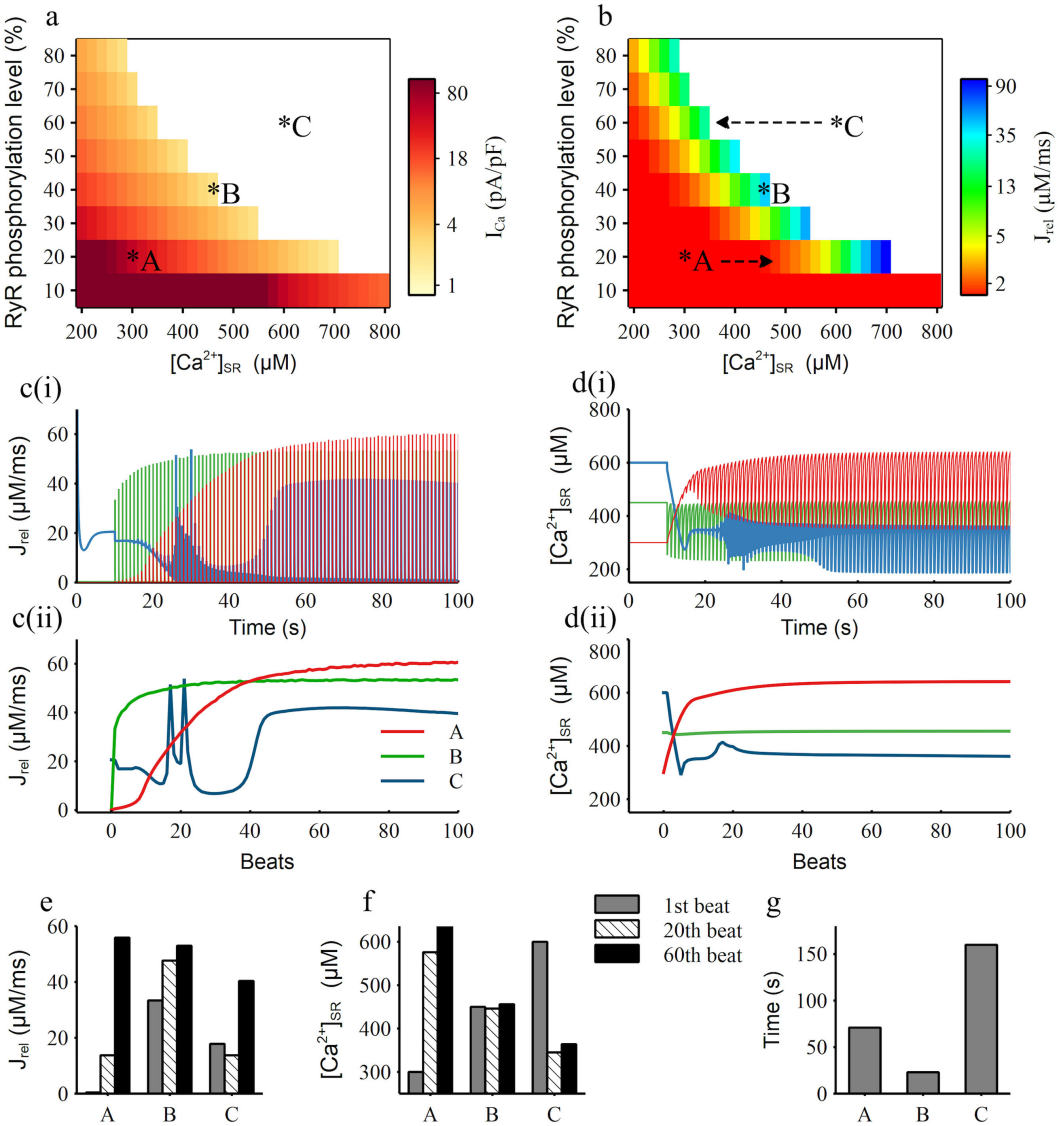
Simulation results of RyR phosphorylation level and [Ca^2+^]_SR_ clamping. **(a,b)** The RyR channel activation threshold map **(a)** and the amplitude of the first RyR release current map **(b)** under corresponding RyR phosphorylation level and [Ca^2+^]_SR_, the top right area shown as the white color means the RyR channel kept open without any stimulus. A, B, C are three representative conditions, which are condition clamping at 20, 40, 60% RyR phosphorylation level and 300, 450, 600 μM [Ca^2+^]_SR_, respectively. Arrows in **(b)** show the condition movement direction after [Ca^2+^]_SR_ unclamped; **(c,d)** The current traces (i) and their maximum values for each beat (ii) of the RyR release current **(c)** and [Ca^2+^]_SR_ **(d)** for the three representative condition marked as A, B, C in **(a)**; **(e,f)** The value of the RyR release current **(e)** and [Ca^2+^]_SR_ **(f)** for the three representative condition for the 1st, 20th, 60th beat; **(g)** The time period for the model to reach a stable RyR release current from the three representative initial conditions.

To further study the main factors that related to the instability of RyR, we carried out the clamping experiments to obtain the RyR activation threshold map under different levels of [Ca^2+^]_SR_ and RyR phosphorylation as shown in Figure 8a. It presents that when [Ca^2+^]_SR_ and RyR phosphorylation level were both low [e.g., [Ca^2+^]_SR_ = 300 μM, RyR phosphorylation level = 20%, marked as condition A in Figure 8a], a large calcium ion current (I_Ca_ = 56pA/pF) was required to induce the SR Ca^2+^ release. With the increase of [Ca^2+^]_SR_ and RyR phosphorylation level, this RyR activation threshold gradually decreased, e.g., a calcium current of only 2.9 pA/pF can induce the SR Ca^2+^ release for condition B [[Ca^2+^]_SR_ = 450 μM, RyR phosphorylation level = 40%], which indicated that RyR was more easily activated and its stability was impaired. When the [Ca^2+^]_SR_ and RyR phosphorylation level increased to a certain extent [e.g., [Ca^2+^]_SR_ = 600 μM, RyR phosphorylation level = 60%, marked as condition C in Figure 8a], RyRs opened immediately after the [Ca^2+^]_SR_ clamp was released and would not close anymore, i.e., it remained open without any Ca^2+^ influx stimulus, which means that the RyR channel was extremely unstable.

Furthermore, according to the RyR release map (Figure 8b), we found that SR Ca^2+^ can release normally (the amplitude of J_rel_ is over 5 μM/ms) only when the level of [Ca^2+^]_SR_ and RyR phosphorylation was in a certain range, which we named as the balance region (shown as the color region from yellow to blue in Figure 8b). For condition A in the left side of the balance region, the [Ca^2+^]_SR_ rise rapidly after releasing the clamp (red line in Figure 8d), which was alike to the model moving from A to the right side as marked in Figure 8b. After around 15 beats, the model moved into the balance region and the SR Ca^2+^ release gradually recovered. For condition C in the right side of the balance region, [Ca^2+^]_SR_ decreased rapidly after unclamping (blue line in Figure 8d), which equaled that the model moving from C to the left as marked in Figure 8b, and the fluctuation of SR Ca^2+^ release became intense. Through the record of RyR release (Figures 8c,e) and [Ca2^+^]SR (Figures 8d,f) through the time course and in the 1st, 20th and 60th beats of condition A, B and C we can clearly see that condition A was accompanied by the rapid increase of J_rel_ and [Ca^2+^]_SR_, condition B had a relatively stable J_rel_ and [Ca^2+^]_SR_, and condition C was noticeably unstable. By recording the time cost for each condition to achieve stability (Figure 8g), which is defined as the situation when variation between two beats is <1%, we can see that condition B firstly became stable, then condition A became stable in about 71 s and condition C took a long time to finally stabilize.

Therefore, we believe that the balance between RyR phosphorylation and [Ca^2+^]_SR_ level is the key factor affecting the stability of the RyR channel. We found that the higher the RyR phosphorylation level is, the narrower and lower range of the concentration of [Ca^2+^]_SR_ is in the corresponding balanced region (Figure 8b). This explains why the high [Na^+^]_i_ can affect the stability of the RyR channel. The elevated [Na^+^]_i_ can lead to relatively high [Ca^2+^]_SR_ while RyR phosphorylation level is also high due to CaMKII hyperactivity, therefore the balance between RyR phosphorylation and [Ca^2+^]_SR_ level was broken, leading to the generation of DADs.

## Discussion

In this study, we built a new CaMKII oxidation module and a refitted CaMKII overexpression module and incorporated them into a mouse atrial model to investigate the arrhythmogenic mechanism of CaMKII overexpression and oxidation. Our simulation results showed that: (1) The overexpression of CaMKII significantly increased the phosphorylation levels of its downstream target proteins, resulting in prolonged AP, increased [Na^+^]_i_, decreased [Ca^2+^]_i_, smaller CaT amplitude, decreased CaT stability and generating more DADs after burst pacing; (2) ROS oxidized CaMKII led to continuous high level of CaMKII activation in both systolic and diastolic phase. The malignant influence of ROS was much more significant with CaMKII overexpression, which considerably disturbed CaT stability and led to persistent DADs that might become the trigger of ectopic activities for AF; (3) The increase in [Na^+^]_i_ could worsen the cell instability, but this may be a secondary cause. Clamping simulations showed that this instability is highly related to the balance between the RyR phosphorylation and [Ca^2+^]_SR_ level, which may be the underlying mechanism of the DAD and AF induced by CaMKII hyperactivity.

### Effect of CaMKII Overexpression and Oxidation on Ca^2+^ Cycling

CaMKII can regulate cardiac excitation-contraction coupling by phosphorylating several target proteins including LTCC, RyR, and PLB. Specifically, this process leads to enhanced LTCC, bringing stronger calcium induced calcium release. RyR phosphorylation enhances RyR's sensitivity to [Ca^2+^]_i_. This, on the one hand, brings stronger SR Ca^2+^ release; on the other hand, lowers the [Ca^2+^]_i_ threshold to induce the SR Ca^2+^ release. The phosphorylation of PLB by CaMKII brings a stronger SR Ca^2+^ reuptake current, leading to a faster Ca^2+^ reuptake process. Overall, CaMKII accelerates the intracellular calcium cycling (from SR Ca^2+^ release to reuptake), and has the effect of strengthening cardiac contraction and relaxation, so that contributes to myocyte adaptation to frequency changes.

In our simulations, when CaMKII was overexpressed, excessive activity of CaMKII led to the increased phosphorylation levels of LTCC, RyR, and PLB. The increased RyR phosphorylation resulted in more Ca^2+^ release from SR and these Ca^2+^ were further transferred out of the myocyte by enhanced forward mode of the I_NCX_. Although increased LTCC phosphorylation led to slightly increased Ca^2+^ influx, this increment was smaller than Ca^2+^ efflux by the I_NCX_, which resulted in a decrease in the total intracellular calcium content at the new steady state ([Ca^2+^]_i_ and [Ca^2+^]_SR_ decrease simultaneously, see Figures 4f,g). Therefore, the role of CaMKII in increasing SR Ca^2+^ release was counteracted by the decrease in [Ca^2+^]_SR_, which could explain why the J_rel_ in the CaMKII-OE model was similar to that in the WT model (Figure 4h). These effects of CaMKII overexpression were amplified by rapid pacing, during which the phosphorylation level of RyR increased, therefore the sensitivity of RyR to Ca^2+^ increased, causing instability of CaT and finally led to extra SR Ca^2+^ release during diastole, which induced DADs.

Simulations with ROS showed that both of the WT and CaMKII-OE models presented a higher activity of CaMKII under oxidative stress. For the WT model, ROS did not have a significant effect on the stability of CaT due to the small basal amount of CaMKII, but only a cardiac strengthen effect (stronger SR Ca^2+^ release and uptake) was observed. On the contrary, serious calcium depletion was found in the CaMKII-OE model with ROS, which reduced the amplitude of CaT and impaired the contraction ability of myocytes. After rapid stimulations, the CaMKII-OE model with ROS showed sustained DADs, indicating a decrease in intracellular Ca^2+^ stability. It is also worth noting that, unlike the overexpression of CaMKII, the effect of ROS on CaMKII activity was slow and sustained. Although ROS did not increase the maximum amount of activated CaMKII, it significantly increased the amount of CaMKII_act_ during diastole. In other words, ROS can activate CaMKII diastolic phase and reduce the response space of CaMKII to rapid stimulations, therefore leading to increased instability of calcium transients after the fast pacing.

### Mechanistic Insight of CaMKII Overexpression and Oxidation in DAD Generation

**Figure 9:**
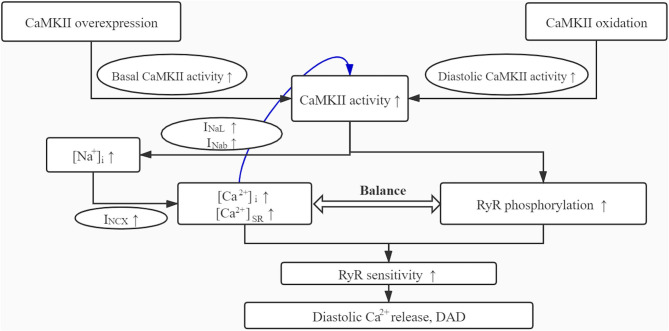
The schematic diagram for the mechanism role of CaMKII overexpression and oxidation in DAD generation.

As shown in the simulations, the overexpression of CaMKII results in the overall increase of basal activity of CaMKII, and the oxidative stress further increases the diastolic activity of CaMKII, whose superposition eventually leads to the continuous hyperactivity of CaMKII (Figure 9). This hyperactivity of CaMKII, on the one hand, directly affects the phosphorylation levels of downstream proteins; on the other hand, it also affects some membrane currents.

Among all membrane currents, the influence of CaMKII on I_NaL_ was considered to play an important role in arrhythmia (Wagner et al., 2006). CaMKII dependent phosphorylation of I_NaL_ increased the Na^+^ influx, resulting in a higher level of [Na^+^]_i_. A large increase in [Na^+^]_i_ was associated with myocyte dysfunction and arrhythmia, which had been shown in previous experimental and simulation studies (Sossalla et al., 2010; Morotti et al., 2014). In this study, our simulation results agreed with their findings. The high level of [Na^+^]_i_ reduced the forward mode of I_NCX_, leading to increased [Ca^2+^]_i_ and [Ca^2+^]_SR_, which played an enhancing role in the activity of CaMKII. This CaMKII-Na^+^ feedback mutually promoted CaMKII activity and [Na^+^]_i_, then enhanced the sensitivity of RyR to Ca^2+^, induced spontaneous SR Ca^2+^ release, and finally led to DAD generation. Experiments found that the application of the sodium channel blocker tetrodotoxin (TTX) can eliminate the above-mentioned arrhythmia, which proved that sodium channel had an important role in the occurrence of DADs. Similarly, our simulation results showed that when [Na^+^]_i_ was clamped at a lower level, most of the DADs caused by CaMKII overexpression and oxidative stress could be eliminated (Figure 7, left), supporting the conclusion of physiological experiments.

Besides, we found that clamping the RyR phosphorylation level to the WT condition could also eliminate the DAD generation (Figure 7, right). Comparing the simulation results of the two clamping methods, it can be found that although the clamping of [Na^+^]_i_ can eliminate DADs, the amplitude of CaTs was still small and the depletion of intracellular calcium ion retained (Figure 7gi). However, the clamping of RyR phosphorylation level substantially limited the loss of intracellular calcium, which supported a larger CaT amplitude and a faster cell recovery after the rapid stimulations (Figure 7gi), which fundamentally reversed the adverse effects of CaMKII overexpression and oxidation.

Our clamping simulations gave a good explanation for this result. It is well-accepted that the generation of DAD is mainly due to the forward model of I_NCX_, which is promoted by the abnormal release of RyR during diastole. Therefore, the stability of the RyR channel plays a key role in the DAD generation. Two main aspects affect the RyR channel. One is the phosphorylation level of RyR and the other is the [Ca^2+^]_SR_, which have greatly influenced each other. By investigating the RyR properties in different clamping conditions, we found that SR Ca^2+^ can release normally only when RyR phosphorylation and [Ca^2+^]_SR_ level reached a balance region, and other clamping conditions always tended to move toward this balance region when they were not in it (Figure 8b). When the model condition was on the left side of the balance region, the RyR channel can hardly open but can move to the balance region rapidly; when the model condition was on the right side of the balance region, the RyR channel was difficult to close, which can be considered as the situation that any small change of Ca^2+^ may lead to the SR Ca^2+^release and it required a long time to return to the balance region (Figure 8g), i.e., the stability of RyR in this condition was extremely low. It can be seen that a higher RyR phosphorylation level are corresponding to a narrower and lower range of [Ca^2+^]_SR_ in the balance region. Since the [Na^+^]_i_ is a key factor affecting [Ca^2+^]_SR_, clamping [Na^+^]_i_ at lower level contribute to lower [Ca^2+^]_SR_ that helps the model moves to the balance region. On the other hand, clamping the phosphorylation level of RyR at a lower level not only leads to a wider range of [Ca^2+^]_SR_ in the balance region, but also induces a larger size of SR Ca^2+^release. This could explain why the stability and release strength of RyR was restored by clamping the RyR phosphorylation level, although the [Na^+^]_i_ was still high.

Therefore, we think that the CaMKII-Na^+^ feedback is an important factor that promotes the cell instability, whereas the high phosphorylation level of RyR due to the hyperactivity of CaMKII may be the leading cause of DADs, which may further become the origin of atrial fibrillations.

### CAMKII May Be a Promising Target for Treating Atrial Fibrillation Patients With Heart Failure

Atrial fibrillation (AF) and Heart failure (HF) are closely related. The incidence rate of AF may increase by 6–8 times in patients with HF (Benjamin et al., 1994), and reduced cardiac output was also observed in AF patients (Medi et al., 2009). The co-existing AF and HF can facilitate each other due to many shared pathophysiological mechanisms, which would considerably increase the mortality and morbidity (Prabhu et al., 2017). However, although AF and HF clearly associate with each other, developing a widely applicable treatment, which benefits both diseases, remains a major challenge. This is due to the increased risk of sudden cardiac death by the application of inotropic drugs (Krell et al., 1986), and arrhythmogenic side effects of traditional antiarrhythmic antagonists on HF patients (Echt et al., 1991). Therefore, it is of great significance to find out the molecular mechanism that can simultaneously benefit the treatment of AF and HF.

Some studies suggested that CaMKII may be a promising target (Swaminathan et al., 2012). It was observed that CaMKII activity and expression significantly increased in AF patients (Tessier et al., 1999; Chelu et al., 2009). And there is also evidence showing CaMKII hyperactivity may contribute to or promote AF (Chelu et al., 2009). On the other hand, CaMKII hyperactivity was found to contribute to HF as well. For example, transgenic CaMKII overexpression mice were exhibited to develop heart failure (Zhang et al., 2003). Also, myocardial infarction causing HF accompanied by CaMKII hyperactivity was observed in mice (He et al., 2011), rabbits (Currie and Smith, 1999) and patients (Sossalla et al., 2010). All evidence suggests that abnormal CaMKII may involve in the development and promotion of both AF and HF. In our simulations, we can observe CaMKII hyperactivity reduced stability of the CaT and produced DADs (Figures 5bii, 6bii), which may develop into the origin of AF. At the same time, our model showed a significant calcium depletion when the model reached the steady state (Figures 5gii, 6gii), i.e., [Ca^2+^]_i_ was greatly reduced with CaMKII hyperactivity, which is related to the reduction of the myocardial contraction force and cardiac output.

Also, more and more evidence shows that cytoplasmic ROS increased in cardiomyocytes under the condition of HF, which has been identified in not only animal models but also patients with systolic and diastolic dysfunction and congestive HF (Heymes et al., 2003; Ijsselmuiden et al., 2008). In our simulations, we found significantly increased CaMKII_act_ during the diastolic period (Figure 6c) under the application of ROS, especially in the CaMKII-OE model. This not only led to sustained DAD generations, but also further reduced the difference of intracellular calcium concentration between diastolic and systolic phase (Figure 6g), resulting in reduced contractile strength, which may lead to the aggravation of heart failure.

Therefore, the overexpression and hyperactivity of CaMKII with extra oxidative stress may be an important mechanism which facilitated both AF and HF. Simulation results suggested CaMKII inhibitors (reduce the total amount of CaMKII or reduce its activity) lead to more stable and larger CaT amplitude, therefore can reduce the incidence of malignant arrhythmia and improve myocardial mechanical function, which may become an important treatment strategy for AF patients with HF.

## Limitation

This study inherited limitations from the baseline model as described in Zhang et al. (2020). Apart from that, this model did not consider the mechanism of sarcolipin undergo CaMKII dependent phosphorylation. Sarcolipin is a key regulator of cardiac SERCA and is expressed widely in atria, which may have a more important effect on SERCA than PLB particularly in atria. The implementation of the sarcolipin may give a more comprehensive understanding on the role of CaMKII in cardiomyocytes. In addition, the ROS in our model only directly affected the CaMKII activity. And the direct influence of ROS on other proteins was not considered. For example, ATP sensitive K^+^ current is an important membrane current that would be greatly affected by the ROS concentration (Bhatnagar, 1997), which is not included in the current model. The energy metabolism system is also absent from our model, albeit it is closely related to the generation of ROS. Finally, the implementation of CaMKII overexpression module in mouse atria may be insufficiently validated due to limited atrial experimental data. However, considering the homology of mouse atrium and ventricle, the difference of CaMKII-OE's effect on electrophysiology between the atrium and ventricle may not be great. Therefore, the proposed model is satisfactory at this stage, and will be further improved with updated experimental data in the future.

## Conclusion

In conclusion, we investigated the arrhythmogenic mechanism of CaMKII overexpression and oxidation by using a mouse atrial cell model, which incorporated a new-built CaMKII oxidation module and a refitted CaMKII overexpression module. Our results showed that the CaMKII overexpression and oxidation had a synergistic role in increasing the kinase activity of CaMKII, leading to CaMKII hyperactivity in both systolic and diastolic phases, which significantly altered the cardiac electrophysiology, calcium cycling, and stability of RyR. The hyperactivity of CaMKII could also induce sustained DADs. We found that the stability of RyR was highly related to the balance between the RyR phosphorylation and [Ca^2+^]_SR_ level, which might be the key mechanism underlying the DAD and AF induced by CaMKII hyperactivity. Finally, we discussed the interrelationship between CaMKII, AF and HF, and CaMKII as a potential target in the treatment for patients with co-existing AF and HF.

## Data Availability Statement

The original contributions presented in the study are included in the article/Supplementary Material, further inquiries can be directed to the corresponding author.

## Author Contributions

WW, SZ, and WS conceived and designed most of the study, performed the simulations and analyses, and wrote most of the manuscript. GL contributed to the figure design and manuscript writing. HZ, YX, and KW supervised the project. All authors contributed to the article and approved the submitted version.

## Conflict of Interest

The authors declare that the research was conducted in the absence of any commercial or financial relationships that could be construed as a potential conflict of interest.
