# Supplementary material for: The Role of CaMKII Overexpression and Oxidation in Atrial Fibrillation—A Simulation Study
Source: Front Physiol. 2020 Dec 18;11:607809. doi: 10.3389/fphys.2020.607809 (PMC7775483; doi:10.3389/fphys.2020.607809)
Supplement: Supplementary file 1 [file Table_1.DOCX]

**Supplementary document I: The chamber-specificity of oxidative CaMKII arrhythmogenicity in mouse**

For showing the chamber-specificity of oxidative CaMKII arrhythmogenicity in mouse, we have integrated the oxidation module into the Morotti model (Morotti et al. 2014) for comparison. As shown in Fig.1a, we can observe quite similar AP morphology for the mouse atrium and ventricle, and additional ROS has little effect on either the atrial or ventricular model. Details of the AP characteristics are shown in the Fig.1b-e, from which we can see that the effect of oxidized CaMKII on the AP is not significantly different.


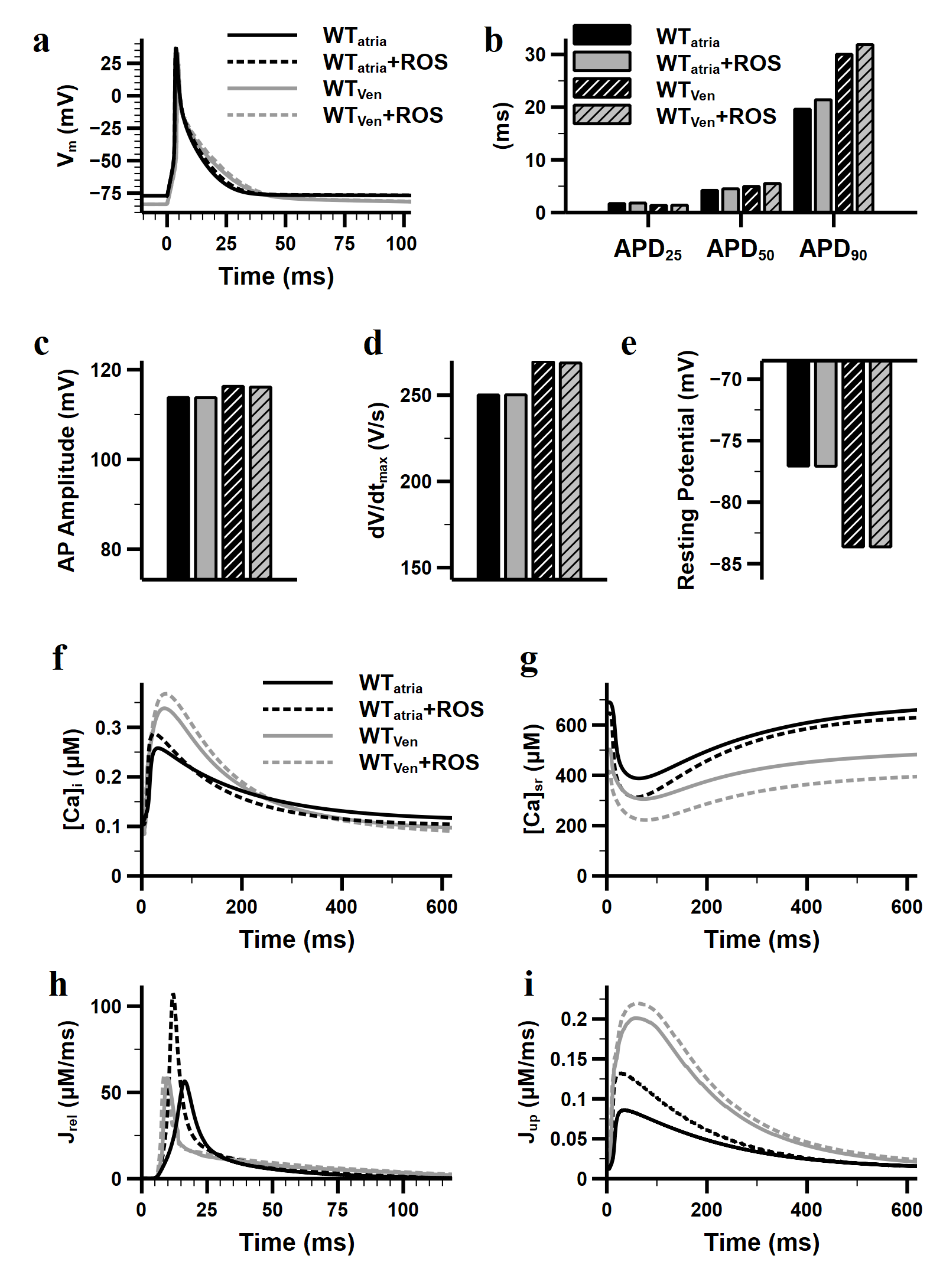


**Supplementary Figure 1. Comparison of the CaMKII oxidation module in the mouse atrial and ventricular model in terms of the characters of action potential (AP) and the calcium cycling process.** Figure shows the superimposed AP (a), recorded APD_25_, APD_50_ and APD_90_ (b), AP amplitude(c), dV/dt_max_(d) and resting potential (e), intracellular Ca^2+^ concentration (f), sarcoplasmic reticulum (SR) Ca^2+^ concentration (g), the SR Ca^2+^ release current (h), the SR Ca^2+^ reuptake current (I) of the WT model and the CaMKII-OE model with and without additional ROS.

On the other hand, additional ROS leads to significant variations on Ca^2+^ cycling in both the atrial and ventricular models. Generally, oxidative CaMKII leads to larger calcium transients in the two chambers (Fig.1f-g), which shows a strengthening effect on the calcium cycling in the two models. However, a significant difference of J_rel_ variation of the two models can be observed (Fig. 1h). We can see that the amplitude of the J_rel_ current in the atrial model substantially increases and the activation of J_rel_ becomes faster under oxidative stress, whereas J_rel_ in the ventricular model does not change very much. This may be due to a slower activation process of J_rel_ current in the atrial model (details about the model differences can be found in (Zhang et al. 2020)). Also, oxidative CaMKII leads to a significantly larger J_up_ in the atrial model compared to the ventricular model (Fig. 1i). In a word, although the AP of mouse atria and ventricles are quite similar to each other, the Ca^2+^ cycling process is substantially different.

References:

Morotti, S., A. G. Edwards, A. D. McCulloch, D. M. Bers, and E. Grandi. 2014. ‘A Novel Computational Model of Mouse Myocyte Electrophysiology to Assess the Synergy between Na+ Loading and CaMKII’. *Journal of Physiology-London* 592 (6): 1181–97. https://doi.org/10.1113/jphysiol.2013.266676.

Zhang, Henggui, Shanzhuo Zhang, Wei Wang, Kuanquan Wang, and Weijian Shen. 2020. ‘A Mathematical Model of the Mouse Atrial Myocyte With Inter-Atrial Electrophysiological Heterogeneity’. *Frontiers in Physiology* 11 (August). https://doi.org/10.3389/fphys.2020.00972.
